# The various faces of vulnerability: offering neurointerventions to criminal offenders

**DOI:** 10.1093/jlb/lsad009

**Published:** 2023-05-06

**Authors:** Sjors Ligthart, Emma Dore-Horgan, Gerben Meynen

**Affiliations:** Willem Pompe Institute for Criminal Law and Criminology, Utrecht University, Utrecht, The Netherlands; Department of Criminal Law, Tilburg University, Tilburg, The Netherlands; Department of Philosophy, VU University, Amsterdam, The Netherlands; Oxford Uehiro Centre for Practical Ethics, University of Oxford, Oxford, UK; Willem Pompe Institute for Criminal Law and Criminology, Utrecht University, Utrecht, The Netherlands; Department of Philosophy, VU University, Amsterdam, The Netherlands

**Keywords:** vulnerability, neurorehabilitation, human rights

## Abstract

In recent years, we have witnessed considerable progress in neurotechnologies that visualize or alter a person’s brain and mental features. In the near future, some of these technologies could possibly be used to change neural parameters of high-risk behavior in criminal offenders, often referred to as neurointerventions. The idea of delivering neurointerventions to criminal justice populations has raised fundamental normative concerns, but some authors have argued that *offering* neurointerventions to convicted offenders could be permissible. However, such offers raise normative concerns too. One prominent worry that is often emphasized in the literature, relates to the vulnerability of convicted offenders in prison and forensic patients in mental health facilities. In this paper, we aim to show that as far as vulnerability is considered relevant within the context of offering medical interventions to offenders, it could contribute to arguments against as well as in favor of these offers.

## I. INTRODUCTION

In recent years, we have witnessed considerable progress in neurotechnologies that visualize or alter a person’s brain and mental features. Shortly, some of these technologies could be used to change neural parameters of high-risk behavior in criminal offenders, often referred to as *neurointerventions*.^1^ Neurointerventions, in general, exert a physical, chemical, or biological effect on the brain to diminish the likelihood of certain forms of criminal offending.^2^ One could think, for instance, of drugs to reduce the libido of sexual offenders with paraphilic disorders,^3^ or the potential use of selective serotonin reuptake inhibitors as a means of reducing impulsive aggression in violent offenders,^4^ but also of emerging neurotechnology. For example, a recent study found that transcranial direct current stimulation (tDCS) reduced self-reported aggression in a forensic population.^5^ And Fuss and collaborators have discussed the possible future use of deep brain stimulation (DBS) as an intervention for sex offenders.^6^

The idea of delivering neurointerventions to criminal justice populations has raised fundamental normative concerns, resulting in a scholarly debate in ethics and law.^7^ Changing brains and altering minds without valid (free and informed) consent is likely to *prima facie* wrong the criminal offender, by infringing a variety of legal and moral rights, such as the right to bodily and mental integrity, the right to freedom of thought, and a right to cognitive liberty. Therefore, it has been argued that the use of neurointerventions can only be permissible with the valid consent of the offender who undergoes the intervention.^8^ Some authors contend that this consent requirement prohibits the imposition of *mandatory* neurointerventions but allows neurointerventions to be *offered* to offenders, for instance in return for parole or probation.^9^

However, such offers raise normative concerns too.^10^ One prominent worry that is often emphasized in the literature, relates to the *vulnerability* of convicted offenders in prison and forensic patients in mental health facilities.^11^ Indeed, offenders’ vulnerability is often invoked, either explicitly or implicitly, in the ethical and legal literature that debates whether offenders can voluntarily choose to undergo a neurointervention when faced with the alternative of serving a long prison sentence or involuntary commitment.^12^ Offers like these may, possibly, sometimes constitute ‘an offer you cannot refuse’.^13^

Life is full of hard choices, and we face difficult offers all the time, without them necessarily being morally or legally wrong. But this may well be different regarding the specific context of offering neurointerventions to criminal offenders. For example, philosophers have argued that when making offers, vulnerability and power asymmetry have the potential to undermine voluntariness and, therefore, the validity of informed consent by the recipient—sometimes referred to as a ‘coercive offer’.^14^ Employing medical interventions, including neurointerventions, on people without their valid consent can be considered a *prima facie* wrong, as it disrespects the person’s autonomous decision-making.^15^ In addition, unlike the general consequences of everyday hard choices, if neurointerventions are applied by the State without the valid consent of the recipient, a range of moral and legal rights is likely to be infringed or even violated. Relatedly, a person’s vulnerability is a relevant factor when determining whether a certain practice qualifies as ‘manipulation’ or ‘exploitation’, behaviors that are commonly considered to be morally objectionable.^16^ Importantly, in the law, too, the vulnerability of convicted offenders—which, according to the European Court on Human Rights, follows from the very nature of their position in criminal justice^17^—appears a relevant factor when determining the permissibility of State interference, especially when the employment of medical interventions in prison is concerned.^18^ The issue of vulnerability has made many appearances in the Court’s case law and is increasingly invoked to ensure context-responsive protection of the rights enshrined in the European Convention of Human Rights (ECHR).^19^

The precise definition and normative value of the concept of vulnerability are under debate.^20^ In this paper, it is not our aim to argue for using vulnerability as a concept in legal and ethical analysis in general. Rather, we aim to show that as far as vulnerability is considered relevant within the context of offering medical interventions to offenders—as is often the case in the literature and the law—it could contribute to arguments *against* as well as *in favor* of these offers.^21^ Although we consider both ethics and the law, our approach is primarily legal, with a focus on the ECHR. The outline is as follows. In Section II, we consider the vulnerability of convicted offenders. Sections III and IV provide an analysis of how the vulnerability could be relevant for arguments against and in favor of offering neurointerventions to criminal offenders, followed by some concluding remarks in Section V.

## II. THE VULNERABILITY OF CONVICTED OFFENDERS

Two central approaches to ‘vulnerability’ are conceivable. The first is to consider it as a universal characteristic of humans as inherently vulnerable beings. This is the approach favored by Martha Fineman who sees vulnerability as ‘*the* primal human condition’ owing to humans’ fragile embodiment.^22^ Alternatively, vulnerability can be approached as a concept that applies to specific, in some way disadvantaged individuals or groups of individuals, emphasizing that particular inequalities, such as power, dependency, and capacity, render some people more vulnerable to harm or exploitation by others.^23^ In cases of vulnerability, usually certain (procedural) safeguards are put in place that should ‘compensate’ the person’s or group’s vulnerability and ensure that their rights are not violated. Note that the vulnerability concept is open to shades of gray and the level of vulnerability may have implications for the extent to which safeguards are required.

The concept of vulnerability has gained increasing prominence within human rights law in recent years. As Roberto Andorno notes, in human rights discourses, ‘the term vulnerability is used to indicate a heightened susceptibility of certain individuals or groups to being harmed or wronged by others or by the state’.^24^ Given that our focus in this paper is on human rights law, in particular the ECHR, our emphasis will be on the individual/group-specific approach to vulnerability in direct relation to offering neurointerventions to convicted offenders. We do, however, acknowledge the value of understanding vulnerability as a general feature of human beings ― at least as a ‘human condition’ of many convicted offenders and forensic patients, especially in theoretical discussions on how to respond to crime and justify punishment, as early adverse life events and one’s social and economic environment are well-known factors that may impact a person’s ability to lead a crime-free life.^25^

Aiming to do justice to both senses of vulnerability, Catriona Mackenzie, Wendy Rogers and Susan Dodds have provided a taxonomy distinguishing between three sources of vulnerability: inherent, situational and pathogenic.^26^ The *inherent* source refers to the general human condition, which is, as indicated, not immediately relevant to our purpose here. *Situational* vulnerability, which is context specific, is, however, significant to our analysis, as the context of detention and other forms of deprivation of liberty render people more vulnerable than those at liberty, e.g., to arbitrary treatment and infringements of fundamental rights, such as the right to human dignity and personal integrity.^27^ Concerns about this kind of situational vulnerability could, for instance, provide an argument against the offering of experimental medical research to detainees.^28^

In addition to situational vulnerability, arising from the criminal justice context, *pathogenic* vulnerability is relevant to our analysis too. It refers, amongst other things, to cognitive problems related to mental disabilities,^29^ which are disproportionately present in convicted offenders in prison. For instance, Seena Fazel and collaborators write that research ‘has consistently shown that prisoners have high rates of psychiatric disorders, and in some countries, more people with severe mental illness are in prisons than in psychiatric hospitals. Despite the high level of need, these disorders are frequently underdiagnosed and poorly treated’.^30^ Among others, substance abuse, attention deficit-hyperactivity disorder (ADHD) and mood disorders have a high prevalence. Recently, interest has increased in the presence of traumatic brain injury (TBI)—brain damage for instance because of a concussion or an anoxic event—in prison populations. In their review, Huw Williams and colleagues conclude that in detainees ‘complicated mild TBI or moderate to severe head injury is prevalent in one to two in 10 people, and another three or four in 10 could have a milder form of TBI. Neuropsychological dysfunction is linked to violence, infractions in prison, poorer treatment gains, and reconviction’.^31^

An even more recent line of research has considered the potential adverse effects of the prison environment on offenders’ mental functioning. For decades, it is known from animal studies that both the enrichment and impoverishment of an animal’s living environment (i.e., both the provision and deprivation of physical, cognitive, and social stimulation and activity)—have an impact on the animal’s brain and functioning.^32^ Also in humans, negative effects of impoverished environments have been reported.^33^ Therefore, it was hypothesized that prison—a deliberately and uniquely impoverished environment—could bring about adverse effects on the neuropsychological functioning of those who are detained. Various studies suggest that such effects of detention exist, reducing capacities for ‘self-control’.^34^ Possibly, this could result in—or deteriorate already present—pathogenic vulnerability. Importantly, such cognitive effects are not only relevant during the time the convicted offender is detained but may also hamper the process of successful rehabilitation after release.^35^ Of note, much about the potential adverse effects of the prison environment on a prisoner’s cognitive functioning is yet unknown. For instance, whereas negative effects have been observed after three to four months of imprisonment, it is unclear whether—and if so, how—neurocognitive capacities would further deteriorate after longer periods. Additionally, it is not clear to what extent these negative effects would restore after release, and how quickly they would be reversible.

In sum, generally, prisoners as a group are characterized by their situational vulnerability, as typical features of detention, such as power asymmetry and dependency, make them extra prone to arbitrary treatment, harm, and infringements of fundamental rights. In addition, many offenders in prison suffer from mental disorders, adding a pathogenic source of vulnerability. Therefore, in actual prison settings inmates are often confronted with a kind of ‘double vulnerability’,^36^ partly arising from imprisonment and the criminal justice system as such and partly from the prevalence of mental disabilities, broadly conceived, so including neuro(psycho)logical problems and potential adverse effects of the prison environment. Importantly, in some (or perhaps many) cases, pathogenic vulnerability may well be reduced, for example, through treating mental illnesses and restoring mental capacities. So, whereas a detainee’s situational vulnerability is typically static and present until the deprivation of liberty ends, the offender’s pathogenic vulnerability is more open to improvement or at least support during imprisonment.

## III. VULNERABILITY AND HUMAN RIGHTS INFRINGEMENTS

Looking first at how human rights might provide grounds against offering neurointerventions to offenders as vulnerable beings, we turn again to the question of consent and vulnerable persons. Informed consent is relevant to the legal protection provided by human rights, as it can prevent rights from being infringed and thus individuals from being wronged. Think of consent to surgery in relation to the right to bodily integrity or consent to a house search by the police in view of  the right to respect for one’s home and private life.^37^ Discussions about the validity of consent vis à vis  arguable infringements of human rights often relate to medical interventions in criminal justice,^38^ such as surgical or chemical castration of sex offenders,^39^ gynecological examination of female detainees,^40^ and forced feeding in prison.^41^ When valid consent exists for a medical procedure, human rights will not normally be infringed. For example, in the case of *Bogumil/Portugal*, a swallowed pellet of cocaine had been surgically removed from the applicant’s stomach. The European Court of Human Rights (ECtHR) considered that if informed consent to this medical procedure had been elicited, as the Government alleged, no issue would have arisen under the prohibition of ill-treatment under Article 3 ECHR.^42^ Put differently, in *Pretty/UK*, the Court emphasized that medical interventions *without* the consent of a mentally competent patient will infringe the right to physical integrity under Article 8 ECHR.^43^

As briefly mentioned in the introduction, employing neurointerventions such as DBS and pharmaceuticals without the valid consent of the criminal offender, is likely to infringe a variety of human rights, such as the right to bodily and mental integrity (Article 8 ECHR),^44^ the prohibition of torture, inhuman and degrading treatment (Article 3 ECHR),^45^ and, possibly, the rights to freedom of thought and freedom of opinion (Articles 9 and 10 ECHR).^46^ Hence, ensuring the valid consent of the offender who undergoes a neurointervention will often be essential to prevent infringements and violations of human rights.

This is where the offender’s vulnerability becomes relevant. In general, valid informed consent requires (1) the disclosure of *appropriate information* (2) to a *competent person* (3) who is permitted to make a *voluntary choice*.^47^ The ECtHR endorses these requirements too, but it is important to realize that the Court has further specified these requirements in its case law to specific situations and contexts, which might not always correspond to the (dominant) philosophical understanding of consent, voluntariness and coercion. For example, in some cases, concerning ‘vulnerable patients’, the Court also requires consent to be explicit.^48^ Furthermore, case law suggests that *situational* vulnerability arising from the deprivation of liberty, could impair voluntariness and, therefore, invalidate consent to medical interventions.^49^

For example, in *R.S./Hungary*, the applicant complained about a violation of the prohibition of ill-treatment under Article 3 ECHR, as he had been catheterized under the control of police officers. The purpose of this medical intervention was to determine whether the applicant, as a road user, had been under influence of alcohol or drugs. Concerning the validity of the applicant’s consent to the catheterization, the ECtHR had ‘doubts as to whether the applicant, being in the hands of the authorities and under their complete control, had any option in practice but to undergo the impugned procedure’.^50^ A similar reasoning has been adopted regarding complaints about gynecological examinations of female prisoners, which has violated the right to the physical integrity of 8 ECHR as well as the prohibition of ill-treatment under Article 3 ECHR.^51^ For instance, in *Juhnke/Turkey*, the applicant complained that she had been subjected to a gynecological examination without valid consent during her time of imprisonment. The Turkish government contended that the examination aimed at protecting prison guards from false allegations of rape and that the applicant had given consent to the employed examination. Concerning the voluntariness of consent, the Court held that, in general:

in certain circumstances, a person in detention cannot be expected to continue to resist submitting to a gynecological examination, given her vulnerability at the hands of the authorities, who exercise complete control over her throughout her detention.^52^

Given the applicant’s vulnerable situation and taking into account other relevant factors including the way she was persuaded to stop resisting and ultimately consent to the gynecological examination, the Court found that ‘it cannot be concluded with certainty that any consent given by the applicant was free and informed. The Court, therefore, considers that the imposition of a gynecological examination on the applicant, in such circumstances, gave rise to an interference with her right to respect for her private life, and in particular her right to physical integrity’.^53^ As this case illustrates, whereas persuasion will normally not adversely affect the voluntariness of consenting decisions,^54^ it might do so in the specific context of offering medical interventions to vulnerable people in prison—at least, according to the ECtHR.

Given the above-mentioned examples of the Court’s case law, Wannes Buelens, Coralie Herijgers and Steffi Illegems have emphasized the difficulty of getting *free* informed consent from people deprived of their liberty. Because of their situational vulnerability, detainees cannot (always) be expected to continue resisting medical interventions that are initiated by the prison authorities. Therefore, Buelens, Herijgers and Illegems suggest a factual presumption that prisoners cannot give voluntary consent to such medical procedures.^55^ In the same vein, regarding surgical castration of sexual offenders in psychiatric hospitals and prisons, the Committee for the Prevention of Torture has warned that consent to these interventions may not always be voluntary due to the situational vulnerability of patients and prisoners:

the Committee considers that the concept of ‘free and informed’ consent is hardly reconcilable with a situation in which the options open to an individual are extremely limited: surgical castration or possible indefinite confinement in a psychiatric hospital.^56^

and:

given the context in which the intervention is offered, it is questionable whether consent to the option of surgical castration will always be truly free and informed. A situation can easily arise whereby patients or prisoners acquiesce rather than consent, believing that it is the only available option to them to avoid indefinite confinement.^57^

In conclusion, all of these examples clearly illustrate the significance of vulnerability to the validity of consent to medical interventions in criminal justice—and thus to legal analyses of whether medical interventions, like neurointerventions, would infringe on human rights. When convicted offenders are deprived of their liberty, their situational vulnerability requires a critical scrutiny of the voluntariness of consenting decisions—on pain of infringing and possibly violating human rights.^58^

Importantly, rights infringements need not always result in rights violations. For example, when chemical castration or tDCS infringes the right to bodily and mental integrity under Article 8(1) ECHR, the infringement might still be justified based on Article 8(2) ECHR—that is, when the neurointervention had a legal basis in domestic law, served a legitimate aim, such as the prevention of crime, and was ‘necessary in a democratic society’. To meet the third requirement, an infringement of Article 8(1) ECHR should correspond to a pressing social need and the means employed must be proportionate to the aims pursued.^59^

For the determination of whether an infringement is proportionate, the national authorities enjoy a certain level of discretion, often referred to as a ‘margin of appreciation’. The discretion States are allowed in this regard can either be ‘wide’, ‘certain’, or ‘narrow’.^60^ The broader the margin that States enjoy, the more discretion they have in finding a ‘fair balance’ between the competing interests at stake.^61^ The breadth of the margin of appreciation varies across individual cases and depends on different factors, such as the nature of the right at stake and the nature and purpose of the infringement. Interestingly, as it appears in some specific cases, the vulnerability of the victim of an alleged violation of human rights could be a relevant factor too, reducing the discretion of the State to restrict human rights and freedoms. For example, as the Court argued in *Alajos Kiss/Hungary* regarding the vulnerability of the mentally disabled to discrimination:

if a restriction on fundamental rights applies to a particularly vulnerable group in society, who have suffered considerable discrimination in the past, such as the mentally disabled, then the State’s margin of appreciation is substantially narrower and it must have very weighty reasons for the restrictions in question.^62^

Likewise, in the case of *J.D. and A/UK*, the Court has stressed that given the need to prevent discrimination against people with disabilities and to foster their full participation and integration in society, the discretion of States in establishing different legal treatments for people with disabilities is ‘considerably reduced’. The particular vulnerability of persons with disabilities would require very weighty reasons to justify such different treatments.^63^ As Alexandra Timmer puts it:

a *bottom line* has emerged: the Court insists that—at the very least—the State should take the particular vulnerability of the persons it is dealing with into account. *Whenever a Government completely omits to consider the particular vulnerability of an individual rights-holder, it will not be able to pass the Strasbourg proportionality analysis.* In other words, paying attention to the particular construction of vulnerability has turned into a procedural requirement.^64^

Given these considerations—which are general and do not relate to the specific context of neurointerventions in criminal justice—it is arguable, from a legal point of view, that the vulnerability of criminal offenders to having their human rights infringed, could narrow the discretion of States to offer neurointerventions in return for parole or probation, requiring weighty or exceptional reasons to justify such offers and the subsequent employment of a specific brain intervention. The extent to which the interests of vulnerable offenders should be prioritized in this sense, is, however, unclear and would probably also depend on the type of vulnerability in question and all other relevant circumstances of the individual case.^65^

## IV. VULNERABILITY AND RIGHTS TO NEUROINTERVENTIONS

### IV.A. Vulnerability and State Obligations

In ethics, it has been argued that a person’s vulnerability could sometimes impose moral obligations on others, such as the State, to reduce vulnerability or to compensate for the effects vulnerability has on a person. For example, Mackenzie, Rogers and Dodds argue that ‘[s]ituational vulnerability gives rise to specific moral and political obligations: to support and provide assistance to those who are currently vulnerable and to reduce the risks of dispositional vulnerabilities becoming occurrent’.^66^ Interventions in response to a person’s vulnerability, they argue, should always aim at enabling or restoring, to the greatest extent possible, the person’s autonomy.^67^ For example, Mackenzie stresses that people with cognitive disabilities, who are vulnerable in the pathogenic sense, ‘are entitled to targeted, publicly funded forms of assistance’.^68^

Likewise, the Committee of Ministers of the Council of Europe recommends that the Member States should ensure mechanisms to protect vulnerable persons with mental disorders—especially those who cannot consent or who may be unable to resist infringements of their human rights.^69^ In this regard, the Committee highlights that in particular situations, individuals with the full cognitive capacity to consent can still be vulnerable because they are subjected to authority, like in prison, or otherwise deprived of their ability to exercise their capacity due to the situation they find themselves in.^70^

In the case law of the ECtHR, too, the vulnerability could sometimes impose positive obligations on the State.^71^ For instance, in the context of prisoners, the ECtHR has argued that people who are deprived of their liberty are in a vulnerable position and that the authorities must protect them.^72^ In some cases, the ECtHR has recognized a positive obligation of the Member States to facilitate and enable prisoners to make progress toward their rehabilitation.^73^

Regarding vulnerable people who suffer from mental disabilities, the ECtHR has emphasized that ‘the mentally ill are in a position of particular vulnerability, and clear issues of respect for their fundamental human dignity arise whenever such persons are detained by the authorities’; their pathogenic vulnerability, in other words, calls for ‘special protection’.^74^ Furthermore, the official Guide on Article 8 ECHR notes:

With regard to the positive obligations that Member States have in respect of vulnerable individuals suffering from mental illness, the Court has affirmed that mental health must also be regarded as a crucial part of private life associated with the aspect of moral integrity. The preservation of mental stability is in that context an indispensable precondition to effective enjoyment of the right to respect for private life.^75^

Depending on their specific features, neurointerventions can *reduce* the vulnerability of convicted offenders, by improving cognitive abilities required for autonomy and for a successful rehabilitation into a free society.^76^ Therefore, Olivia Choy, Farah Focquaert and Adrian Raine have argued that categorically denying offenders the benefit of safe and effective neurointerventions would probably increase the vulnerability of an already vulnerable group, by excluding (or restricting) the options of successful rehabilitation and achieving a crime-free life.^77^ Rather, they contend:

if an adequate level of safety and effectiveness can be guaranteed, our analysis argues in favor of offering various options to offenders (e.g., incarceration or biological interventions) as this maximally respects offenders’ autonomy, bodily integrity, and mental integrity, increases the motivation for and effectiveness of the sanction in question, and has the potential to reduce the criminogenic effects of imprisonment.^78^

Denying defendants the opportunity to make their *own* decisions regarding the acceptance of some forms of mind-altering interventions—just because of their alleged vulnerability—can be considered outright paternalistic,^79^ which is often regarded (morally) problematic,^80^ also by the ECtHR.^81^ Both lawyers and ethicists have highlighted the potential risks of the vulnerability language in law and policy, which may be used as a moral justification for social control and behavioral regulation and could lead to discrimination, stereotyping, and unjustified forms of paternalism.^82^ As Marc Blitz has emphasized, the situational vulnerability of prisoners and detainees should not too easily become an argument or excuse to deny convicted offenders the opportunity of using novel technologies to voluntarily modify their minds for rehabilitation.^83^

Given the positive obligations the ECtHR recognizes regarding vulnerable prisoners and patients, it is arguable that there could be a *prima facie* duty on the part of the State to provide—mirrored by a right of vulnerable offenders to receive—the available means, also if they are neurotechnological in nature, to help restore or preserve mental capacities required for autonomy. Recently, it has been argued that convicted offenders do indeed have a right to enhance their mental capabilities with the use of neurotechnology. Generally, two arguments can be distinguished: the first relates to a legal *right to mental self-determination* and the second to a moral *right to neurorehabilitation*. In principle, both could serve as a substantial argument against categorically denying vulnerable offenders the possibility to participate in risk-reducing neurointerventions, e.g., in return for parole or probation. We briefly consider these two arguments below.

### IV.B. A Right to Mental Self-Determination

Considering emerging neurotechnologies that enable both to intervene in people’s minds and detect mental activity, Christoph Bublitz and Reinhard Merkel have argued for a human right to mental self-determination,^84^ sometimes also referred to as a right to cognitive liberty.^85^ Generally, the scope of this right is twofold: it encompasses a negative and a positive dimension. In the negative dimension, the right protects freedom *from* significant, non-consensual interferences in our minds. This part of the right could be invoked, for example, against the State applying neurointerventions to convicted offenders without their valid consent.^86^ In the positive dimension, the right to mental self-determination protects the freedom *to* self-determine one’s mental features. It covers a right ‘to alter one’s mind, not only by one’s natural capacities but also with the help of neuro tools from pharmaceuticals to brain stimulation’.^87^

Likewise, referring to the work of Joel Feinberg,^88^ Marc Blitz highlights that the sovereignty we have over our bodies entails both negative and positive freedoms, such as the freedom *not* to have surgery against one’s will and the freedom *to* have surgery if one voluntarily chooses it. According to Blitz, this is arguably true too regarding the sovereignty we have over our minds: ‘We might, for example, have a negative right against having our minds coercively altered with drugs or other neurointerventions but also a positive right to voluntarily change our brain chemistry in this way, at least in some circumstances’.^89^ In the same vein, Marcello Ienca and Roberto Andorno have argued that the moral right to ‘cognitive liberty’ also entails a positive freedom of being able to act in a way to take control over one’s own mental life.^90^

Under the ECHR, a right to mental self-determination could be grounded in different rights and freedoms. For instance, some have argued that the *negative* right against mind-altering interventions by others can be derived from the qualified right to mental integrity (Article 8 ECHR) and, possibly, also from the absolute right to freedom of inner thought (Article 9 ECHR).^91^ Furthermore, the *positive* right to self-determine and alter one’s mental features seems suitable to be derived from the general right to self-determination, which is covered by the broader right to respect for private life under Article 8 ECHR.^92^ In addition, it has been argued that the positive dimension of a right to mental self-determination could also be grounded in the right to freedom of thought.^93^ Whether and how exactly a right to mental self-determination should be incorporated into the established framework of European human rights is a matter of present debate. A thorough discussion of this topical question would exceed the scope of the present paper. But we do want to highlight the observation of Marcello Ienca in a recent report for the Committee on Bioethics of the Council of Europe, that there is general agreement on the basic premises of a right to mental self-determination (which he calls cognitive liberty).^94^ As a consequence, we would be inclined to argue that the positive dimension of the right to mental self-determination deserves serious attention in normative debates on whether neurointerventions could or should be offered to vulnerable offenders.

### IV.C. A Right to Neurorehabilitation

In recent work, one of us has argued that offenders have a (non-absolute) moral right to ‘neurorehabilitation’.^95^ More specifically, offenders have a moral right to the offer of safe and affordable neurointerventions when these would be part of the most effective package for facilitating their rehabilitation.^96^ Three moral bases for a right to neurorehabilitation have been identified, *two* of which emerge precisely because and when offenders are put in a position of situational and pathogenic vulnerability following conviction—that is, (1) offenders are entitled to be offered neurorehabilitation as a means to counteract the debilitating side-effects of many of our punishment practices; and (2) a moral right to neurorehabilitation can be derived from offenders’ moral right to hope for renewed liberty.

The first moral basis can be seen as appealing to the State’s moral obligation to reduce or avoid creating *pathogenic* vulnerability in offenders through punishment. This obligation is implicitly recognized in the law too. Article 3 ECHR, for example, prohibits the meting out of degrading treatment or punishment—i.e., a punishment that shows a lack of respect for human dignity or that arouses fear, anguish, or inferiority.^97^ European and U.S. case law has ruled that ‘violence to our societal notions of the intrinsic worth and dignity of human beings’^98^ is indeed occasioned when offenders are subjected to an ‘impoverished regime’ that risks undermining their mental functioning and hence their chances of reforming themselves.^99^ Moreover, the ECtHR holds that, in certain circumstances, providing life prisoners with a real opportunity for rehabilitation may require that ‘they be enabled to undergo treatments or therapies—be they medical, psychological or psychiatric—adapted to their situation to facilitate their rehabilitation’.^100^ Likewise, under the right to liberty under Article 5 ECHR, the ECtHR considers that when detention is (predominantly) justified on grounds of dangerousness and public protection, rather than retribution, prisoners should be offered real opportunities to rehabilitate themselves—such as suitable therapy—to make progress through the prison system and become eligible for parole.^101^ As Adriano Martufi stresses: ‘In the absence of such offending-behavior programs, a deprivation of liberty based exclusively on the presumed dangerousness of the offenders would amount to ‘arbitrary detention’, within the meaning of Article 5(1)(a) ECHR’.^102^

Reducing or avoiding the induction of pathogenic vulnerability in offenders—such as by restoring mental disabilities and effectively countering the degenerative impact of existing penal practices—might sometimes require the delivery of (safe and affordable future) neurointerventions. Emphasizing the value of an individual’s resilience in forestalling penal degeneration, Dore-Horgan has pointed to how several pharmaceutical interventions show promise for promoting resilience in their recipients.^103^ When such interventions would be part of the most effective package for preserving offenders’ ability to maintain a normal or near-normal level of functioning in the face of carceral impoverishment, it can be argued that offenders have a moral right to be offered them.^104^

The second identified moral basis for a right to neurorehabilitation can be seen as appealing to the State’s moral obligation to preserve offenders’ hope for an end to their *situational* vulnerability. Again, this moral obligation is implicitly recognized in law as well. In *Vinter/UK,* for example, the ECtHR judged that it would be incompatible with human dignity ‘to forcefully deprive a person of his freedom without at least providing him with the chance to someday regain that freedom’.^105^

In the case of some offenders, preserving a genuine and tangible prospect for rehabilitation and release might require the provision of neurorehabilitation. Offenders’ moral right to hope for rehabilitation and release is probably best understood as a right to hope for their achievement ‘at *acceptable costs,* in terms of effort, to [themselves]—not a right to hope for rehabilitation through gargantuan effort’.^106^ In situations where conventional rehabilitative measures prove or can be expected to prove ineffective in facilitating rehabilitation and/or where rehabilitation without neurointervention would be a gargantuan task, then offenders have a moral right to avail of adjunctive neurorehabilitative treatment that promises to deliver the more effective rehabilitative package, so it has been argued.^107^

Of note, a *legal* right to neurorehabilitation cannot yet be derived from the ECHR. The ECtHR has maintained, for example, that Article 3 ECHR ‘cannot be construed as imposing on the authorities an absolute duty to provide prisoners with rehabilitation (…) programmes and activities’.^108^ At present, the ECHR does not guarantee a right to rehabilitation or neurorehabilitation per se.^109^ Nonetheless, the Court has maintained that several other non-legally binding instruments to which it attaches considerable importance, like the European Prison Rules, emphasize that efforts must be made by the prison authorities to promote the reintegration and rehabilitation of prisoners.^110^ As discussed above, the Court also presupposes that convicted offenders, including life prisoners, be provided with conditions and a prison regime that enable them to make progress toward their rehabilitation such that they might one day be eligible for parole or conditional release, e.g., through the use of medical interventions. It seems that the established framework of European human rights thus implicitly recognizes that offenders ought not be made or kept vulnerable or be denied the effective opportunity to rehabilitate and, thus, all hope for the alleviation of their situational vulnerability following conviction and punishment, even if it falls short of recognizing (neuro)rehabilitation as a right.^111^ Yet, to the extent that the avoidance of the above states of affairs might in the medium-term future be best served by providing offenders with neurointerventions, there is a *moral* argument in favor of offering them. And we contend that this moral argument deserves greater attention than it has hitherto been afforded within the normative debate.

## V. CONCLUDING REMARKS

In the ethical and legal debate about the acceptability of offering neurointerventions to offenders, vulnerability is often invoked. Usually, vulnerability features in arguments against offering this possibility. In this paper, however, we aimed to show that the vulnerable status of offenders furnishes arguments both for and against offering neurointerventions to offenders. On the one hand, it can be argued that the State ought to refrain from offering neurointerventions to offenders lest the latter’s situational and/or pathogenic vulnerability might undermine their ability to validly consent to these interventions. On the other hand, it can be argued that offenders have a moral and/or legal right to mental self-determination. In other words, their vulnerability should not automatically result in denying them the opportunity to make their own choices (paternalism). In addition, it could be argued that there is a moral right to rehabilitation that obliges the State to make safe and effective neurointerventions available to this population. Put differently, based on their vulnerability an argument can be made for a right to be offered neurointerventions, at least under certain conditions.

As said, our aim in this paper has been to document and draw attention to these opposing vulnerability-based arguments. We have not offered a proposal as to how these opposing arguments should be weighed against each other when deciding whether and when neurointerventions could or should be offered to offenders, and a discussion of this would require more extended treatment than we can afford it here. However, let us make three related comments concerning this quickly.

In discerning whether and when neurointerventions might permissibly be offered to offenders, it might be instructive to look to the existing guidance and regulations surrounding *clinical research* involving prisoners. This latter context has many parallels with the context of neurointerventions. Here, we similarly have concerns about the possibility of eliciting valid consent to clinical trial participation from this situationally and/or pathogenically vulnerable population; and hence concerns about the appropriateness of offering participation to these individuals. We also have the concern that denying prisoners the opportunity to participate in clinical research denies them something to which they have a right—in this case, the right to receive equivalent healthcare (and equivalent health-enhancing opportunities) to that enjoyed by the non-carceral population.^112^ An examination of the available legal and ethical guidelines in this comparable context might thus serve as a starting point for discerning whether and when offering neurointerventions to vulnerable offenders might be permissible.

A preliminary examination of European laws and regulations concerning research involving prisoners reveals some guidance that might feasibly be appropriated to the context of offering neurointerventions. One piece of guidance, for example, maintains that research conducted in dubiously consensually circumstances must, if it is to be permissible, have the potential to be of ‘direct benefit’ to participants or entail ‘minimal risks and minimal burden’,^113^ understood as ‘slight and temporary impact on the health of the person concerned’.^114^ Another piece of guidance urges that research participants be ‘permitted to opt out at any time’.^115^ And a further stipulation is that the research to which prisoners are invited must be approved by a ‘board of ethics’.^116^ We suggest that it would be fruitful to interrogate whether similar restrictions might serve to protect the vulnerable targets of neurointerventions.

In addition, it is important to involve those who are—or have been—incarcerated in the process of decision-making too. Not only in the application of future neurotechnologies but also in the design and development of these techniques,^117^ which may be crucial for the question of whether and how the techniques can be responsibly applied. Research involving those with first-hand experience of imprisonment is likely to be valuable in this respect.^118^

## FUNDING

This paper was funded by the Dutch Research Council (NWO Vici grant VI.C.201.067). The contribution of EDH was also funded by the Wellcome Trust (Grant number 217709/Z/19/Z).

## Footnotes

1 J. Ryberg, Neurointerventions, Crime, and Punishment (2020); N.A. Vincent, T. Nadelhoffer & A. Mc Cay (eds.), Neurointerventions and the Law (2020).

2 D. Birks & T. Douglas, Treatment for Crime (2018), 2.

3 L. Forsberg, *Anti-Libidinal Interventions and the Law*, 21 HRLR 384 (2021).

4 C. Chew et al., *Biological Interventions for Crime Prevention,* in treatment for crime (D. Birks and T. Douglas eds., 2018) at 22–25. For the protocol of a systemic study that has recently been commissioned to investigate the benefits of administering SSRIs to impulsively violent offenders, see T. Butler et al., *Sertraline Hydrochloride for Reducing Impulsive Behaviour in Male, Repeat-Violent Offenders (ReINVEST): Protocol for a Phase IV, Double-Blind, Placebo-Controlled, Randomised Clinical Trial,* 11 BMJ (2021), doi: 10.1136/bmjopen-2020-044656.

5 C.S. Sergiou et al., *Transcranial Direct Current Stimulation Targeting the Ventromedial Prefrontal Cortex Reduces Reactive Aggression and Modulates Electrophysiological Responses in a Forensic Population*, 7 Biol Psychiat: CNNI 95 (2022). See also R. Knehans et al, *Modulating Behavioural and Self-Reported Aggression with Non-Invasive Brain Stimulation: A Literature Review*, 12 Brain Sci. 200 (2022).

6 Cf. J. Fuss et al., *Deep brain stimulation to reduce sexual drive*, 40 J Psychiatry Neurosci 429 (2015).

7
*Supra* note 1–4.

8 For discussion see T. Douglas, *Criminal Rehabilitation Through Medical Intervention: Moral Liability and the Right to Bodily Integrity*, 18 J. Ethics 101 (2014).

9 J. Pugh*, Coercion and the Neurocorrective Offer*, in Birks & Douglas *supra* note 2.

10 Pugh *supra* note 9; Ryberg *supra* note 1.

11 O. Choy, F. Focquaert & A. Raine, *Benign Biological Interventions to Reduce Offending*, 13 Neuroethics 29 (2020); J. Pugh & T. Douglas, *Neurointerventions as criminal rehabilitation: An ethical review*, in The Routledge Handbook of Criminal Justice Ethics (J. Jacobs & J. Jackson, eds. 2016); P. Kellmeyer, N. Biller-Andorno & G. Meynen, *Ethical tensions of virtual reality treatment in vulnerable patients* 25 Nat. Med. 1185 (2019). See also B.E. McDermott, *Coercion in Research: Are Prisoners the Only Vulnerable Population?*, 41 J. Am. Acad. Psychiatry Law 8 (2013); L. Pasquerella, *Confining choices: should inmates’ participation in research be limited?* 23 Theor Med Bioeth 519 (2002).

12 It is sometimes also invoked when concerns are voiced about the ‘mere’ or unconditional offer of neurointervention within liberty-restricted environments such as the carceral setting: W. Green, *Depo-Provera, Castration, and the Probation of Rape Offenders: Statutory and Constitutional Issues*, 12 univ dayton law review 1(1986).

13 G. Meynen, *Brain-based Mind Reading in Forensic Psychiatry*, 4 JLB 311. (2017).

14 J. McGregor, *Undue Influence as Coercive Offers in Clinical Trials* in Coercion and the State (D.A. Reify & W.J. Riker eds., 2008); D. Zimmerman, *Coercive Wage Offers*, 10 Philos Public Aff 121 (1981); J. Feinberg, The Moral Limits of the Criminal Law: Volume 3: Harm to Self (1989). This view is, however, contested, see eg A. Wertheimer & F.G. Miller, *There are (STILL) no coercive offers*, 40 J. Med. Ethics 592 (2014).

15 A.K. Martin, N. Tavaglione, S.A. Hurst: ‘Resolving the Conflict: Clarifying Vulnerability in Health Care Ethics.’ Kennedy Institute of Ethics Journal 2014, 24 (1): pp. 51–72.

16 M. Zwolinski & A. Wertheimer, The Stanford Encyclopedia of Philosophy (E.N. Zalta ed., Summer 2017 Edition); R. Noggle, *The Ethics of Manipulation*, in The Stanford Encyclopedia of Philosophy (E.N. Zalta ed., Summer 2020 Edition).

17
*Panovits/Cyprus*, ECtHR Dec. 11, 2008, 4268/04, at 68; A.H.E. Morawa, *Vulnerability as a Concept of International Human Rights Law,* 6 JIRD 139 (2003), at 143; A. Timmer, *A Quiet Revolution: Vulnerability in the European Court of Human Rights*, in Vulnerability: Reelections on a New Ethical Foundational for Law and Politics (M.A. Fineman & A. Grear eds., 2013), at 154. See also the Commentary on Recommendation Rec(2006)2 of the Committee of Ministers to member states on the European Prison Rules.

18 S. Ligthart, Coercive Brain-Reading in Criminal Justice: An Analysis of European Human Rights Law (2022), at 241.

19 C. Heri, Responsive Human Rights: Vulnerability and the Ecthr (2021).

20 A. Timmer et al., *The Potential and Pitfalls of the Vulnerability Concept for Human Rights,* 39 neth quart human rights 190 (2021); Heri, *supra* note 19; A.K. Martin, N. Tavaglione, S.A. Hurst*, Resolving the Conflict: Clarifying Vulnerability in Health Care Ethics*, 24 Kennedy Institute of Ethics Journal 51 (2014); N. Tavaglione et al., *Fleshing out Vulnerability*, 29 Bioethics 98 (2015); B.Clark & N. Preto, *Exploring the concept of vulnerability in health care*, 190 Cmaj 308 (2018), doi: 10.1503/cmaj.180242.

21 See also S. Ligthart & G. Meynen, *Offering Neurotechnology to Defendants: On Vulnerability, Voluntariness, and Consent*, in Neuroscientific Perspectives of Vulnerable Defendants in the Courtroom (H. Wishart & C.M. Berryessa, eds. 2023). We focus on neurointerventions, but some parts of our analysis will also be relevant to offering other technologies or interventions, such as ‘neurotechnological monitoring’. In addition, we realize that our analysis of the faces of vulnerability, although motivated by concerns about offering neurointerventions, may well be relevant to other far-reaching decisions convicted offenders—and possibly other vulnerable groups—may have to make.

22 M.A. Fineman, *Equality and Difference: The Restrained State* 66 alabama law review 609 (2015), at 614.

23 C. Mackenzie, W. Rogers & S. Dodds, *Introductio*n in Vulnerability: New Essays in Ethics and Feminist Philosophy (C. Mackenzie, W. Rogers & S. Dodds eds., 2014), at 6; L. Peroni & A. Timmer, *Vulnerable groups: The promise of an emerging concept in European Human Rights Convention law*, 11 I•CON 1056 (2013), at 1058.

24 R. Andorno, *Is Vulnerability the Foundation of Human Rights?*, in Human Dignity of the Vulnerable in the Age of Rights (A. Masferrer & E. García-Sánchez eds., 2016), at 258. See also Peroni & Timmer *supra* note 23, at 1076.

25 See eg F Coppola, Humanizing Prison through Social Neuroscience, in The Routledge Handbook of the Philosophy and Science of Punishment (G. Focquart, E. Shaw & B. Waller Eds. 2021), at par. 16.3.

26 Mackenzie, Rogers and Dodds *supra* note 23.

27 Morawa *supra* note 17, at 143; H.S. Aasen, *Vulnerability and Autonomy: Competing Ideas in Human Rights*, in Health and Human Rights (B. Toebes et al. eds., 2022), at 233; Timmer *supra* note 17, at 154; *Kurt/Turkey*, ECommHR Dec. 5, 1996, 24,276/94, at 201.

28 See Pasquerella *supra* note 11.

29 Cf. *Alajos Kiss/Hungary*, ECtHR May 20, 2010, 38,832/06, at 42.

30 S. Fazel et al., *Mental health of prisoners: prevalence, adverse outcomes, and interventions*, 3 Lancet Psychiat 871 (2016).

31 W.H. Williams, *Traumatic brain injury: a potential cause of violent crime?*, 5 Lancet Psychiat 836 (2018).

32 K. Coleman, J. L.Weed & S.J. Schapiro, *Chapter 4 - Environmental Enrichment for Animals Used in Research*, in Animal Models for the Study of Human Disease (P.M. Conn ed., 2013); M.J Renner & M.R. Rosenzweig, Enriched and Impoverished Environments (1987); H. van Praag, G. Kempermann & F.H. Gage, *Neural consequences of enviromental enrichment,* 1 Nat. Rev. Neurosci. 191 (2000).

33 K.M. Volkers & E.J. Scherder, *Impoverished environment, cognition, aging and dementia*, 22 Rev. Neurosci. 259 (2011).

34 J. Meijers et al., *Reduced Self-Control after 3 Months of Imprisonment; A Pilot Study*, 9 Front Psychol (2018); R. Umbach, A. Raine, & N.R. Leonard, *Cognitive Decline as a Result of Incarceration and the Effects of a CBT/MT Intervention: A Cluster-Randomized Controlled Trial,* 45 Crim Justice Behav 31 (2018). See also J. Meijers, J.M. Harte & G. Meynen, *The Neuropsychology of Executive Functioning and Violence*, in Forensic Neuroscience and Violence (J.M. Fabian ed., in press); Coppola *supra* note 25; F. Coppola, *The Brain on Solitude: An (other) Eighth Amendment Challenge to Solitary Confinement*, 6(1) JLB 184 (2019), with further references.

35 S. Ligthart et al., *The Relevance of Neuropsychology for Social Rehabilitation: A Human Rights Perspective*, in Social Rehabilitation & Criminal Justice (F. Coppola & A. Martufi eds., in press); S. Ligthart et al., *Neuropsychological research in light of the European Convention on Human Rights*, 10 NJECL 287 (2019).

36 Choy, Focquaert & Raine *supra* note 11, at 33.

37 Cf. D.J. Harris et al., Harris, O’Boyle, and Warbrick: Law of the European Convention on Human Rights (2018), at 238.

38 W. Buelens, C. Herijgers & S. Illegems, *The View of the European Court of Human Rights on Competent Patients’ Right of Informed Consent* 23 Eur. J. Health Law 481 (2016).

39 CPT/Inf (2012)6, point 145; Forsberg *supra* note 3.

40
*Juhnke/Turkey*, ECtHR May 13, 2008, 52,515/99; *Y.F./Turkey*, ECtHR July 22, 2003, 24,209/94.

41 P. Jacobs, *Force-feeding of prisoners and detainees on hunger strike* (Intersentia 2012), 68–72.

42
*Bogumil/Portugal*, ECtHR Oct. 3, 2008, 35,228/03, at 71.

43
*Pretty/UK*, ECtHR Apr. 29, 2002, 2346/02, at 63, 67. Cf. *V.C./Slovakia*, ECtHR Nov. 8, 2011, 18,968/07, at 105. Under Article 8 ECHR, the right to physical integrity is part of the broader right to respect for private life: K. de Vries, *Right to Respect for Private and Family Life*, in Theory and Practice of the European Convention on Human Rights (P. van Dijk et al. eds., 2018), at 667–734.

44 S. Ligthart et al., *Closed-Loop Brain Devices in Offender Rehabilitation: Autonomy, Human Rights, and Accountability*, 30 Camb C Healthc Ethic 669 (2021); J.N. Craig, Incarceration, Direct Brain Intervention, and the Right to Mental Integrity – a Reply to Thomas Douglas’, 9 Neuroethics 107 (2016).

45 L. Kirchmair, *Objections to Coercive Neurocorrectives for Criminal Offenders – Why Offenders’ Human Rights Should Fundamentally Come First*, 38 Crim. Justice Ethics 19 (2019).

46 J.C. Bublitz, *Freedom of Thought in the Age of Neuroscience* 100 arsp 1 (2014); Report of the Special Rapporteur on the promotion and protection of the right to freedom of opinion and expression, A/73/348 (2018), at 23.

47 N. Eyal, *Informed Consent*, in The Stanford Encyclopedia of Philosophy (E.N. Zalta ed., Spring 2019 Edition); J. Pugh, Autonomy, Rationality, and Contemporary Bioethics (2020), Ch 6.

48 Buelens, Herijgers & Illegems *supra* note 38. Cf. *Panovits/Cyprus*, ECtHR Dec. 11, 2008, 4268/04, 68: ‘given the vulnerability of an accused minor and the imbalance of power to which he is subjected by the very nature of criminal proceedings, a waiver by him or on his behalf of an important right under Article 6 can only be accepted where it is expressed in an unequivocal manner after the authorities have taken all reasonable steps to ensure that he or she is fully aware of his rights of defence and can appreciate, as far as possible, the consequence of his conduct’.

49 Ligthart *supra* note 18.

50
*R.S./Hungary*, ECtHR July 2, 2019, 65,290/14, at 65.

51
*Y.F./Turkey*, ECtHR July 22, 2003, 24,209/94 (Article 8 ECHR); *Yazgül Yılmaz/Turkey*, ECtHR Feb. 1, 2011, 36,369/06 (Article 3 ECHR).

52
*Juhnke/Turkey*, ECtHR May 13, 2008, 52,515/99, at 76. Cf., differently: *Dvořáček/Czech Republic* Nov. 6, 2014, 12,927/13.

53
*Juhnke/Turkey*, ECtHR May 13, 2008, 52,515/99, at 77.

54 Feinberg, *supra* note 14, at 189.

55 Buelens, Herijgers & Illegems *supra* note 38, at 488, 506.

56 CPT/Inf (2007)32, at 109.

57 CPT/Inf (2012)6, at 145.

58 Ligthart *supra* note 18, Ch. 8.

59
*Slivenko/Latvia*, ECtHR Oct. 9, 2003, 48,321/99, at 113; S. Ligthart, *Coercive Neuroimaging, Criminal Law and Privacy: A European Perspective*, 6 JLB 289 (2019).

60 L. Lavrysen, *System of Restrictions*, in Theory and Practice of the European Convention on Human Rights (P. van Dijk et al. eds., 2018), at 328.

61 J. Gerards, *Pluralism, Deference and the Margin of Appreciation Doctrine*, 17 European Law Journal 80 (2011), at 105–107.

62
*Alajos Kiss/Hungary*, ECtHR May 20, 2010, 38,832/06, at 42.

63
*J.D. and A/UK,* ECtHR Oct. 24, 2019, 32,949/17, 34,614/17, at 89; *Murray/The Netherlands*, ECtHR April 26, 2016, 10,511/10, at 106.

64 Timmer *supra* note 17, at 164–165 (original emphasis).

65
*Id.*

66 Mackenzie, Rogers & Dodds *supra* note 23, at 8.

67
*Id.* at 9.

68 C. Mackenzie, *The Importance of Relational Autonomy and Capabilities for an Ethics of Vulnerability*, in Mackenzie, Rogers & Dodds *supra* note 23, at 53.

69
Recommendation Rec (2004)10 of the Committee of Ministers to Member States concerning the Protection of the Human Rights and Dignity of Persons with Mental Disorder, Art. 7.

70
Explanatory Memorandum to Recommendation Rec (2004)10, Art. 7.

71 Peroni & Timmer *supra* note 23, at 1076; Timmer *supra* note 17, at 165–167.

72
*Keenan/UK*, ECtHR Apr. 3, 2001, 27,229/95, at 91; Salman/Turkey, ECtHR June 27, 2000, 21,986/93, at 99.

73 S. Meijer, *Rehabilitation as a Positive Obligation*, 25 Eur. J. Crime Crim. Law Crim. Justice 145 (2017).

74
*M.S./UK*, ECtHR May 3, 2012, 24,527/08, at 39; *Renolde/France*, ECtHR Oct. 16, 2008, 5608/05, at 109.

75
*Guide on Article 8 of the European Convention on Human Rights*, August 2021, at 129. Cf.

76 Choy, Focquaert & Raine *supra* note 11, at 34; C.H. de Kogel, *More Autonomous or more Fenced-in? Neuroscientific Instrument and Interventions in Criminal Justice*, 12 Neuroethics 43 (2019).

77 Choy, Focquaert & Raine supra note 11, at 33.

78
*Id*. Also: F. Focquaert, K. Van Assche & S. Sterckx *Offering Neurointerventions to Offenders With Cognitive-Emotional Impairments: Ethical and Criminal Justice Aspects*, in Vincent, Nadelhoffer & McCay *supra* note 1.

79 Cf. L. Pasquerella, *supra* note 11, at 523: ‘twenty-two states have taken further steps to ban the use of inmates in biomedical research altogether. While these paternalistic measures are designed to protect a population that is vulnerable due to their environment from the abuses of the past, the effect in many instances has been to deny prisoners access to the only available treatment for their diseases.’

80 Mackenzie, Rogers & Dodds *supra* note 23, at 15: ‘Many contributors to the volume, however, are also mindful of the fact that social policy discourses of vulnerability and protection can be used to justify paternalistic and coercive forms of state intervention that generate pathogenic forms of vulnerability. For this reason, many contributors argue that duties of protection must be guided by the overall aim of promoting autonomy.’

81
*V.C./Slovakia*, ECtHR Nov. 8, 2011, 18,968/07, 114. Cf. *Jehovah’s Witnesses of Moscow/Russia***,** ECtHR June 10, 2010, 302/02, at 136: “The freedom to accept or refuse specific medical treatment, or to select an alternative form of treatment, is vital to the principles of self-determination and personal autonomy. (…) However, for this freedom to be meaningful, patients must have the right to make choices that accord with their own views and values, regardless of how irrational, unwise or imprudent such choices may appear to others.”

82 Timmer et al., *supra* note 20; S.Y. Kim*., Les vulnérables: evaluating the vulnerability criterion in Article 14 cases by the European Court of Human Rights*, 41 Legal Studies 617 (2021); Mackenzie, Rogers & Dodds *supra* note 23, at 15, 16.

83 M.J. Blitz, *Extended Reality, Mental Liberty, and State Power in Forensic Settings*, 13 (2022) 173 AJOB-N, at 175.

84 J.C. Bublitz & R. Merkel, *Crimes Against Minds: On Mental Manipulations, Harms and a Human Right to Mental Self-Determination*, 8 Crim Law Phil 51 (2014).

85 Farahany, The Battle For Your Brain (2023); J.C. Bublitz, *My Mind Is Mine!? Cognitive Liberty as a Legal Concept*, in Cognitive Enhancement (W. Hildt & A.G. Franke eds., 2013); M. Ienca, Common human rights challenges raised by different applications of neurotechnologies in the biomedical field, Report commissioned by the Committee on Bioethics of the Council of Europe (2021), at 55.

86 J.C. Bublitz, ‘“The Soul is in the Prison of the Body”—Mandatory Moral Enhancement, Punishment & Rights against Neuro-rehabilitation’, in Birks & Douglas *supra* note 2.

87 J.C. Bublitz, *Cognitive Liberty or the International Human Right to Freedom of Thought*, in Handbook of Neuroethics (J. Clausen & N. Levy eds., 2015), at 1317. Also: Ienca *supra* note 85, at 55.

88 Feinberg *supra* note 14.

89 Blitz *supra* note 83, at 174.

90 M. Ienca & R. Andorno, *Towards new human rights in the age of neuroscience and neurotechnology*, 13 LSSP (2017), at 11. See also J.J. Fins, *The Unintended Consequences of Chile’s Neurorights Constitutional Reform: Moving beyond Negative Rights to Capabilities*, Neuroethics (2022, online first).

91 S. Michalowski, *Critical Reflections on the Need for a Right to Mental Self-Determination*, in The Cambridge Handbook of New Human Rights: Recognition, Novelty, Rhetoric (A. von Arnauld, K. von der Decken & M. Susi eds., 2020); S. Ligthart et al., *Is Virtually Everything Possible? The Relevance of Ethics and Human Rights for Introducing Extended Reality in Forensic Psychiatry*, 13 AJOB-N 144 (2021), at 151–152.

92
*Parrillo/Italy*, ECtHR Aug. 27, 2015, 46,470/11, at 153. Apart from a general right to self-determination, the ECtHR has also acknowledged a right to ‘informational self-determination’ under Article 8 ECHR: *Satakunnan Markkinapörssi Oy and Satamedia Oy/Finland*, ECtHR June 27, 2017, 931/13, at 137.

93 Bublitz *supra* note 87, at 1318; Ienca *supra* note 85, at 56; Blitz *supra* note 83, at 175.

94 Ienca *supra* note 85, at 56.

95 E. Dore-Horgan, *Do Criminal Offenders Have a Right to Neurorehabilitation?*  Crim. Law Philos. (2022), doi.org/10.1007/s11572-022-09630-y.

96
*Id.* at 1.

97
*Pretty/United Kingdom* ECtHR Apr. 29, 2002, 2346/02, at 52; *Svinarenko and Slyadnev v. Russia* ECtHR July 17, 2014, 32,541/08 and 43,441/08, at 115.

98
*Laaman/Helgemoe,* U.S. D.N.H., July 1, 1977, 437F. Supp. 269, at 323.

99
*Harakchiev and Tolumov/Bulgaria*, ECtHR July 8, 2014, 15,018/11 and 61,199/12, at 264–266.

100
*Murray/The Netherlands*, ECtHR Apr. 26, 2016, 10,511/10, at 109.

101
*Sy/Italy*, ECtHR Jan. 24, 2022, 11,791/20; *Klinkenbuß/Germany*, ECtHR Feb. 25, 2016, 53,157/11, at 47; *James, Wells en Lee/UK,* ECtHR Sept. 18, 2012, 25,119/09, 57,715/09 and 57,877/09, at 217–218.

102 A. Martufi, *The paths of offender rehabilitation and the European dimension of punishment*, 25 Maastricht J. Eur. Comp. Law 672 (2018), at 678.

103 For some of the empirical evidence, see A.K. Friedman et al., *KCNQ Channel Openers Reverse Depressive Symptoms via an Active Resilience Mechanism,* 7 Nat. Commun. 11671 (2016); A. Tan et al., *Effects of the KCNQ Channel Opener Ezogabine on Functional Connectivity of the Ventral Striatum and Clinical Symptoms in Patients with Major Depressive Disorder*, 25 Mol. Psych 1323 (2020).

104 Dore-Horgan *supra* note 95, at 8.

105
*Vinter and others/the United Kingdom* ECtHR July 9, 2013, 66,069/09, 130/10 and 3896/10, at 113.

106 Dore-Horgan *supra* note 95 at 13 (original emphasis).

107
*Id.* at 13–14.

108
*Harakchiev and Tolumov/Bulgaria*, ECtHR July 8, 2014, 15,018/11 and 61,199/12, at 264.

109
*Murray/the Netherlands,* ECtHR Apr. 26, 2016, 10,511/10, at 103.

110
*Harakchiev and Tolumov/Bulgaria,* ECtHR July 8, 2014, 15,018/11 and 61,199/12, at 264. In addition, the European Prison Rules also clearly impose a duty upon on States to facilitate (mental) healthcare.

111 The potential consequences of accepting neurointerventions in terms of earlier release are a topic of debate, see also the Introduction—we will not further address them in this paper.

112 B.S. Elger, *Research involving Prisoners: Consensus and Controversies in International and European regulations,* 22 Bioethics 224 (2008).

113 Additional protocol to the Convention on Human Rights and Biomedicine concerning Biomedical research (2005), Art. 15.

114
*Id.* Art 17.

115 Elger *supra* note 112, at 227.

116 European Committee for the Prevention of Torture and Inhuman or Degrading Treatment or Punishment (CPT), Health care services in prisons, *Extract from the 3rd General Report of the CPT, published in 1993*, Art. 48.

117 See Kellmeyer, Biller-Andorno & Meynen *supra* note 11. Their argument concerns virtual reality, but the basic ideas regarding responsible use are also relevant to the application of neurotechnologies.

118 N. Knack, J.A. Chandler & J.P. Fedoroff, *A qualitative study of forensic patients’ perceptions of quasi-coercive offers of biological treatment,* 38 Behav Sci Law 135 (2020).

